# Value of combining biological age with assessment of individual frailty to optimize management of cancer treated with targeted therapies: model of chronic myeloid leukemia treated with tyrosine kinase inhibitors (BIO-TIMER trial)

**DOI:** 10.1186/s12885-024-12415-2

**Published:** 2024-05-30

**Authors:** Mélanie Casile, Gilles Albrand, Clément Lahaye, Benjamin Lebecque, Joévin Besombes, Céline Bourgne, Bruno Pereira, Sandrine Saugues, Caroline Jamot, Eric Hermet, Marc G. Berger

**Affiliations:** 1 Biological Hematology Department, CHU Clermont-Ferrand, Clermont-Ferrand, France; 2 Geriatric Evaluation and Management unit, Antoine Charial Hospital, Francheville, Lyon, France; 3 EA 7453 CHELTER, University of Clermont Auvergne, Clermont-Ferrand, France; 4 Biostatistics Unit, Clinical Research and Innovation Department, CHU Clermont-Ferrand, Clermont-Ferrand, France; 5 Biological Resources Centre – Auvergne, CHU Clermont-Ferrand, Clermont-Ferrand, France; 6 Clinical Hematology Department, CHU Clermont-Ferrand, Clermont-Ferrand, France; 7 Unité mobile de Gériatrie, CHU Clermont-Ferrand, Clermont-Ferrand, France

**Keywords:** Biological Age, Individual Frailty, Chronic myeloid leukemia, Tyrosine kinase inhibitors

## Abstract

**Background:**

In the era of targeted therapies, the influence of aging on cancer management varies from one patient to another. Assessing individual frailty using geriatric tools has its limitations, and is not appropriate for all patients especially the youngest one. Thus, assessing the complementary value of a potential biomarker of individual aging is a promising field of investigation. The chronic myeloid leukemia model allows us to address this question with obvious advantages: longest experience in the use of tyrosine kinase inhibitors, standardization of therapeutic management and response with minimal residual disease and no effect on age-related diseases. Therefore, the aim of the BIO-TIMER study is to assess the biological age of chronic myeloid leukemia or non-malignant cells in patients treated with tyrosine kinase inhibitors and to determine its relevance, in association or not with individual frailty to optimize the personalised management of each patient.

**Methods:**

The BIO-TIMER study is a multi-center, prospective, longitudinal study aiming to evaluate the value of combining biological age determination by DNA methylation profile with individual frailty assessment to personalize the management of chronic myeloid leukemia patients treated with tyrosine kinase inhibitors. Blood samples will be collected at diagnosis, 3 months and 12 months after treatment initiation. Individual frailty and quality of life will be assess at diagnosis, 6 months after treatment initiation, and then annually for 3 years. Tolerance to tyrosine kinase inhibitors will also be assessed during the 3-year follow-up. The study plans to recruit 321 patients and recruitment started in November 2023.

**Discussion:**

The assessment of individual frailty should make it possible to personalize the treatment and care of patients. The BIO-TIMER study will provide new data on the role of aging in the management of chronic myeloid leukemia patients treated with tyrosine kinase inhibitors, which could influence clinical decision-making.

**Trial registration:**

ClinicalTrials.gov, ID NCT06130787; registered on November 14, 2023.

## Background

Chronological age is a limiting factor in the administration of anti-cancer treatments, but the development of targeted therapies and advances in overall management have made it possible to extend the age limit for prescribing them. The evolution of personalised medicine partly explains the reduction in mortality rates since the end of the 1990s. However, therapeutic efficacy and side effects vary from one patient to another, depending not only on age but also on the patient’s general condition and comorbidities. A better characterisation of individual biomarker for ageing is a key challenge for personalised medicine.

Ageing is classically determined by chronological age, which only partially reflects physiological ageing. The only tools used in practice to assess the individual impact of ageing are based on surveys to detect frailty [1] by assessing the functional impact of age (quality of life, cognitive capacity, mobility, autonomy), mainly developed and used by geriatricians [2]. Age-related individual frailty has been shown to influence cancer management and progression [3]. However, in the era of targeted therapies, these tools are not suitable for all patients, as most of them were developed with elderly populations [1–5]. Furthermore, they are only suitable for individual frailty that has already present but not for early frailty or the risk of developing frailty in a pathological context such as cancer.

Complementary tools to assess individual biological and functional ageing would appear to be of particular relevance [5]. The relationship between chronological age (in years) and biological age (BA) (the ageing of cells/tissues) and their relationship with the development or the treatment of cancer remains poorly defined, especially for targeted therapies. Cellular ageing is a complex and multifactorial process [5, 6] related to pathologies (Parkinson’s disease, cardiovascular disease, cancer, etc.).

Recently, biomarkers of ageing called epigenetic clock have been described. These clocks are based on DNA methylation (DNAm) and enabled an accurate estimation of BA for any tissue. In fact, changes in DNA methylation during ageing [7–10] are correlated with the risk of developing age-related pathologies and mortality. In many cancers, the DNAm clock is altered, usually towards accelerated ageing. Moreover, an association between accelerated BA and the risk of cancer has been described [9, 11], but the relationship between biological ageing and the characteristics of the cancer and/or its evolution during treatment or tolerance to treatment remains poorly understood.

Chronic myeloid leukemia (CML) appears as a model to study these relationship. Firstly, it is a model of personalised medicine with the first targeted therapy (tyrosine kinase inhibitors), and thus has the longest track record of use. Moreover, the management of CML and the assessment of therapeutic response are standardized [12]. Unlike other cancers, CML is considered to have a minor influence on the biological functions altered by ageing, thus avoiding a major confounding factor [13]. Furthermore, there is no clear relationship between CML development and predisposition factor such as clonal hematopoiesis of undetermined significance (CHIP) in the course of ageing. Finally, some preliminary results suggest the existence of age-related DNA methylation abnormalities common to all patients, regardless of age. This could be linked to the emergence and amplification of the CML clone and possibly to an individual predisposition [14]. Lastly, some tyrosine kinase inhibitors (TKI) as nilotinib and ponatinib can induce side effects considered as aging-related diseases, for example cardio-vascular toxicity. These properties make CML as a particularly suitable model for studying the influence of age-related biological features other than CHIP.

Few studies have analysed the relationship between the DNA methylation profile and individual frailty in cancer patients treated with targeted therapy. Its influence on response and, in particular, tolerance to treatment remains to be studied. In fact, the influence of chronological age is tending to decrease due to the greater efficacy and better tolerance of targeted therapies, but some patients present significant side effects that may be related to ageing.

The aim of the BIO-TIMER protocol is therefore to assess the value of combining the determination of BA using the DNA methylation clock with the assessment of individual frailty in order to personalise the management of CML patients treated with TKI.

## Methods and analysis

### Study design

The BIO-TIMER study is a multi-center, prospective, longitudinal study aiming to evaluate the value of combining BA determination with individual frailty assessment to personalize the management of CML patients treated with TKI. This study has been registered on Clinicaltrials.gov (NCT06130787). A maximum of 321 patients are expected to be enrolled. The study was started in November 2023 with a 2-year enrolment period and an estimated completion date by November 2028.

### Coordination and participating institutions

The CHU Clermont-Ferrand is the sponsor and is responsible for coordination, trial management, data management, trial monitoring and statistical analysis. This multicenter study will be conducted in 16 sites in France. The list of the study sites is available on https://clinicaltrials.gov/study/NCT06130787.

The CRB-Auvergne (BB-0033-00039) coordinates the collection of biological samples. All activities have been certified according to the NF S 96–900 standard since July 2013, ISO 9001 certified since July 2020 and ISO 20,387 certified since October 2022 thus ensuring the quality of biological resources, harmonization of professional practices and compliance with current regulations.

### CML observatory

The clinico-biological data from patient follow-up will be recorded in an already existing database: the CML observatory. The aim of the CML observatory is to establish the largest possible real-life cohort collecting long-term follow-up of a maximum number of CML patients in France, in order to carry out observational studies [15]. Any patient with a recent or previous diagnosis of CML may be included. Each patient’s clinical and laboratory data are recorded after obtaining informed consent from the patient. The record sheet has been adapted to the follow-up of patients with CML. Data recording is retrospective and prospective. In the case of the BIO-TIMER study, the data will be collected prospectively. The patient’s characteristics, treatments (dosage, treatment changes and their reasons), and laboratory results (particularly BCR::ABL transcript quantification) will be recorded. Some variables, such as prognostic scores are calculated automatically.

### Study objectives and endpoints

#### Main objective and endpoint

The main objective of the study is to evaluate the value of combining BA determination with individual fragility assessment to personalize the management of CML patients treated with TKI, in terms of tolerances.

The first primary endpoint is the difference between BA and chronological age (of the CML clone and non-malignant cells in the same patient) expressed as a difference and as a percentage (ratio).

The second endpoint is the individual frailty assessed using G-CODE score, Rockwood Frailty Scale and Charlson Comorbidity Index.

And the last endpoint is the TKI tolerance in patients with CML assessed by actual dose received, TKI discontinuation due to intolerance, TKI discontinuation due to adverse events, TKI changes due to intolerance and number of TKI lines.

#### Secondary objectives

The secondary objectives are:

- Describe the distribution of individual clinical frailty levels in a cohort of patients with chronic-phase CML at diagnosis time.
- Describe tolerance criteria (actual dose received, TKI discontinuation due to intolerance, TKI discontinuation due to adverse events, TKI changes, number of TKI lines and treatment sequences).
- Evaluate the value of combining BA determination with individual frailty assessment to personalize the management of CML patients treated with TKI, in terms of therapeutic response assessed using the BCR::ABL transcript and quality of life assessed using the EORTC QLQ-C30 questionnaire.

### Participant eligibility

The inclusion and non-inclusion criteria are presented in Table 1. Newly diagnosed chronic phase - CML patients will be eligible for the protocol.

**Table 1:** Section criteria

Inclusion criteria	Non-inclusion criteria
Female or male 18 years of age or older	CML in accelerated or blast phase
CML-CP diagnosis (chronic phase) with confirmation of a Philadelphia chromosome (Ph1) made no more than 3 months (90 days) prior to inclusion. A cryptic Ph1 chromosome must be confirmed by FISH. Criteria must meet the CML chronic phase definition criteria: - < 15% blasts in peripheral blood and bone marrow - < 30% blasts + promyelocytes in peripheral blood and bone marrow - < 20% basophils in peripheral blood, - Platelet count ≥ 100 × 109/L (≥ 100,000/mm3)	
BCR ::ABL1 transcript quantifiable by quantitative RT-qPCR	Refusal to participate
1st-line treatment with tyrosine kinase inhibitors	
No treatment with tyrosine kinase inhibitors or hydroxyurea prior to first blood sampling (at diagnosis)	Treatment started before inclusion
Signature of informed consent for the CML Observatory and signature of informed consent for the BIO-TIMER protocol	Patients under guardianship, curatorship, deprivation of liberty or safeguard of justice
Read and understand French	Pregnant or breastfeeding woman
Persons affiliated to a social security system	

### Intervention

Eligible patients will be enrolled following a consultation with an investigator, after checking that they meet the inclusion and exclusion criteria (and signing an informed consent).

The specific needs of the study are to provide a biological sample and to assess individual frailty and quality of life longitudinally.

Blood samples will be collected at diagnosis, 3 months and 12 months after treatment initiation. Optionally, a bone marrow sample taken at diagnosis will be retained. This will be used to compare the BA between the blood and bone marrow compartments.

An assessment of individual frailty and quality of life will be performed at diagnosis, 6 months after treatment initiation, and then annually for 3 years.

Pseudonymized blood samples will be stored by every sites and then sent to the CRB-Auvergne (France) for analysis. Blood samples will be analyzed to assess BA.

The risks or constraints for subjects participating in the study appear to be minimal, as the only intervention is blood tests.

### Determination of biological age and evaluation of individual frailty

DNA methylation profile analysis is currently the most robust method for assessing BA. DNA methylation clocks mainly use data from HM450K chips (Illumina), which cover the majority of CpG islands. However, these have been replaced by the recently validated EPIC BeadChip kit, despite the loss of some targets [16]. Based on the results obtained with the EPIC BeadChip kit, BA will be determined using the Horvath [10], Hannum [7] and DNAmPhenoAge [8] algorithms, which combine the methylation profile and certain biological assays. In addition, next generation sequencing will be performed to assess the presence of mutations of CHIP and measurement of telomere length will be carried out.

These analyses will be carried out at the time of diagnosis, prior to any treatment, in order to characterise the DNA methylation profile of malignant cells. They will also be carried out 3 months and 12 months after the start of treatment, when the residual BCR::ABL1/ABL1 transcript is low. This will enable the DNA methylation profile of non-malignant cells to be described.

Individual frailty will be assessed using the Rockwood score [17] and the G-CODE questionnaire [18]. The Rockwood score is a measure of frailty based on clinical judgement. The scale ranges from 1 (very good physical condition) through 7 (complete functional dependence) to 9 (at the end of life). The rise of one category on the scale significantly increases the risk, in the medium term, of death and admission to a health establishment. The scale is simple to use and can be easily administered in a clinical setting, enabling care to be adapted and communication with the patient or the various clinical staff to be adapted. The G-CODE defines the minimum geriatric information to be collected at the time of inclusion of patients in clinical trials. It is used to describe elderly cancer patients and to standardise the collection of geriatric data in therapeutic trials but could be applied to younger patients. It consists of the following 7 parts: Social assessment: living alone or support requested to stay at home; Functional autonomy: Activities of Daily Living questionnaire and short Instrumental Activities of Daily living questionnaire; Mobility: Timed Up and Go test; Nutrition: weight loss during the past 6 months and body mass index; Cognition: Mini-Cog test; Mood: mini-Geriatric Depression Scale; Comorbidity: updated Charlson Comorbidity Index.

### Study procedures and participant timeline

The overview of study assessments and procedures are presented in Table 2.

**Table 2:** Data collection schedule

	DIAGNOSTIC	ACTIVE TREATMENT PHASE				
	INCLUSION	M3 *(+/- 8 days)*	M6 *(+/- 8 days)*	M12 *(+/- 10 days)*	M24 *(+/- 10 days)*	M36 *(+/- 10 days)*
Information and consent	X					
**Clinical evaluation**						
Medical history and current comorbidities	X					
History of the disease	X					
Weight and height (BMI calculation)	X	X	X	X	X	X
Rockwood score **	X		X	X	X	X
EORTC QLQ C30	X		X	X	X	X
G-CODE	X		X	X	X	X
Treatment tolerance		X	X	X	X	X
**Biological evaluation**						
Biological collection (blood)	X	X		X		
Optional biological collection (marrow)	X					
Albumin, C-reactive protein, creatinine, creatinine clearance*, hemoglobin	X	X	X	X	X	X
**Tumor evaluation**						
Evaluation of tumor response (hematological, cytogenetic, molecular, plasma assay, BCR::ABL mutation when tested)		X	X	X	X	X

*According to the Cockroft-Gault formula and the CKD-EPI equation

**If carried out by a clinical research associate or a nurse, the assessment must be validated by a geriatrician

### Sample size

The sample size in order to study the relationship between BA, individual frailty and tolerance to TKIs will be estimated according to Hanlon P. et al. [19], Huan-Tze L. et al. [20] and the data from CML Observatory.

Considering 45% frail and pre-frail patients, an effect size greater than 0.5 (moderate to large according to Cohen’s rules of thumb) between BA and individual frailty can be highlighted with 200 patients, for a two-sided type I error at 0.01 (correction due to multiple comparisons) and statistical power above 80%. An interim analysis is planned at inclusion of 100 patients to estimate statistical power according to observed effect sizes and 95% confidence intervals.

Furthermore, given the data reported in the literature [21] and data from CML Observatory, it seems reasonable to expect 35% TKI intolerance. Thus, an enrolment of 321 patients will enable us to identify a 15% absolute difference in intolerance (treated as censored data) for a two-sided type I error of 5% and statistical power of 80%, which correspond to a reasonable hazard ratio of at least 1.4.

An interim analysis is planned at inclusion of 100 patients to re-estimate statistical power according to observed effect sizes and 95% confidence intervals for the relationship between BA and individual frailty.

### Data analysis

Continuous data will be expressed using mean and standard deviation or median and interquartile range, according to their statistical distribution. The normality assumption will be studied using the Shapiro-Wilk test.

The relationship between chronological age and BA will be investigated using correlation and concordance coefficients (Lin for continuous data and Cohen’s kappa for categorical data), Bland and Altman plot and approaches based on non-linearity assumptions. The difference between BA and chronological age will be expressed as a difference and as a percentage (ratio) to assess the relevance of these two modalities.

The relationship between BA, difference between BA and chronological age and frailty will be analyzed using correlation coefficients (Pearson or Spearman, depending on the statistical distribution) and mediation analysis.

A detailed description of frailty will be carried out. First, frailty will be treated as an ordinal variable. Then, frailty will be categorized according to statistical distribution and to data reported in the literature. Likewise, age (BA and chronological age) will be considered as a continuous variable and then as a categorical variable with groups defined according to their clinical relevance and statistical distribution. The relationships will be analyzed using the Chi-square test or Fisher’s exact test (categorical x categorical) and the analysis of variance or Kruskal-Wallis test if the assumptions of the analysis of variance are not met (categorical x continuous).

For longitudinal analyses, mixed models will be used to analyze the relationship between BA, difference between BA and chronological age, and temporal changes of level of frailty, taking into account between and within patient variability (patient as random-effect). The evolution of frailty will be also assessed by trajectory modelling. The relationship between trajectories and the difference between BA and chronological age will be analyzed using analysis of variance or Kruskal-Wallis test. Multivariate analyses will be carried out to take into account confounding factors selected according to univariate results and to their clinical relevance. A particular attention will be paid to multicollinearity. Center will be considered as random-effect. The results will be expressed with effect sizes and 95% confidence intervals. Sensitivity analyses will be proposed in order to study the statistical nature of the missing data and to propose the most appropriate imputation method.

The relationships between BA, difference between BA and chronological age, individual frailty and quality of life will also be investigated.

Tolerance criteria (actual dose received, discontinuation of TKI due to intolerance, adverse events, TKI changes and number of TKI lines) will be the subject of detailed descriptive analysis. Then, marginal Cox model with time-dependent variables and joint models for longitudinal data will be used to analyze the impact of BA, difference between BA and chronological age, and individual frailty on tolerance to TKIs and therapeutic response. The proportional hazards hypothesis will be tested using the Schoenfeld test and residual plot. Death will be treated as a competitive risk using Fine and Gray model. Multivariate analyses will be performed to take account of possible confounding factors (e.g. sex, age, Sokal and ETLS scores, hemoglobin, platelets, leukocytosis, splenomegaly, type of treatment).

Statistical analyses will be carried out using Stata software (version 15, StataCorp, College Station) for a two-tailed type I error at 5% applying when appropriate a Sidak’s correction for multiple comparisons.

### Data management and monitoring

Data on individual frailty and quality of life, specific to the BIO-TIMER study, will be recorded on an eCRF (Ennov Clinical software). Data from the usual follow-up of CML patients will be recorded into the CML Observatory. The users with access to the data will be the investigators, the geriatricians, the clinical research associates, the project leaders and the biostatisticians. They are authorized professionals and are subject to professional secrecy. The investigator will ensure the accuracy, completeness, and consistency of the data recorded (pseudonymized patient data) and of the provision of answers to data queries. A clinical research associate mandated by the sponsor will perform regular monitoring reviews. The objectives will be to ensure the proper conduct of the study, the recording of the data generated in writing, and its documentation, storage and reporting, in accordance with the legislative and regulatory provisions in force. The follow-up reports will ensure traceability.

### Consideration of risk of bias

The study will be offered to newly-diagnosed patients meeting the inclusion criteria, regardless of age or comorbidities, in order to avoid any selective bias on potential patient frailty. Patient management and treatment with TKI will not be affected by the study. The multicentric organization enables patients to be included prospectively from several regions, with different age distributions, and will ultimately provide a representation of the French CML cohort at diagnosis and will reduce a potential center-effect. Comparison of this sub-cohort with the CML Observatory cohort (> 1300 patients) will enable us to verify its representativeness. Attrition bias should be minimal, as patients will be followed according to the recommendations of the European Leukemia Net (ELN), which should limit management variability and lost to follow-up. In addition, a regular data check with the use of queries will be proposed in order to limit missing data. In addition, sensitivity analyses will be proposed to investigate the statistical nature of missing data (completely at random or not) and to propose, if necessary, the most appropriate imputation method. Finally, an interim analysis will be performed every 100 patients to assess the distribution of individual frailty subgroups and analyze the relationship between individual frailty, BA and chronological age.

### Trail status

The BIO-TIMER trial is currently recruiting. Participant recruitment began in November 2023 and recruitment is expected to finish in November 2025. The approved protocol is version 1, 15 June 2023.

## Discussion

In the era of targeted therapies, aging has had an influence on cancer management, due to their higher efficiency and their lower toxicity. But tolerance strongly varies from one patient to another depending of the comorbidities and sometimes without any other disease. The evaluation of individual frailty should allow to personalise treatment and patient care. However, geriatric assessment tools are insufficient to evaluate patients in this context. The discovery of DNA methylation clocks, considered the best tools for evaluating BA, could represent a valuable aid for the evaluation of these patients.

The relationship between biological aging and individual fragility is still relatively understudiedand mainly concerns general populations and is limited in terms of chronological age categories [22–26] or the capacity to predict mortality [27]. Very recently, it has been reported that accelerated aging could precede the onset of detectable frailty [28].

The BIO-TIMER study is the first prospective trial elaborated to test the value of combining BA determination with individual frailty assessment to personalise the management of a targeted therapy treated cancer with CML patients treated with TKI as model.

These assessments could be predictive of adverse events, opening up new prospects for the management of tolerance to targeted therapies. We will study in particular the relationship between cardiovascular events secondary to taking nilotinib, which can occur in patients without any cardiovascular risk factor (personal observation).

They could also provide new perspectives for assessing the risk of good or poor response, particularly in younger patients, if changes in cellular aging influence therapeutic response. Finally, these combined datas could also make it possible to propose a new prognostic score for CML.

The BIO-TIMER study will thus provide new data on the role of aging in the management of CML patients treated with TKI, which could influence clinical decision-making.

## Data Availability

No datasets were generated or analysed during the current study.

## Abbreviations

BA: Biological Age
CHIP: Clonal Hematopoiesis of Indeterminate Potential
CML: Chronic Myeloid Leukemia
TKI: Tyrosine Kinase Inhibitors
