# Correction: Value of combining biological age with assessment of individual frailty to optimize management of cancer treated with targeted therapies: model of chronic myeloid leukemia treated with tyrosine kinase inhibitors (BIO-TIMER trial)

**DOI:** 10.1186/s12885-024-12592-0

**Published:** 2024-07-05

**Authors:** Mélanie Casile, Gilles Albrand, Clément Lahaye, Benjamin Lebecque, Joévin Besombes, Céline Bourgne, Bruno Pereira, Sandrine Saugues, Caroline Jamot, Eric Hermet, Marc G. Berger

**Affiliations:** 1 Biological Hematology Department, CHU Clermont-Ferrand, Clermont-Ferrand, France; 2 Geriatric Evaluation and Management unit, Antoine Charial Hospital, Francheville, Lyon, France; 3 EA 7453 CHELTER, University of Clermont Auvergne, Clermont-Ferrand, France; 4 Biostatistics Unit, Clinical Research and Innovation Department, CHU Clermont-Ferrand, Clermont-Ferrand, France; 5 Biological Resources Centre – Auvergne, CHU Clermont-Ferrand, Clermont-Ferrand, France; 6 Clinical Hematology Department, CHU Clermont-Ferrand, Clermont-Ferrand, France; 7 Unité mobile de Gériatrie, CHU Clermont-Ferrand, Clermont-Ferrand, France

**Correction: BMC Cancer 24, 661 (2024)**

10.1186/s12885-024-12415-2.

Following publication of the original article [1], it was reported that the third author, Clément Lahaye, was erroneously omitted from the author group. Clément Lahaye is affiliated with Unité mobile de Gériatrie, CHU Clermont-Ferrand, Clermont-Ferrand, France.

The author group above has been updated and the original article [1] has been corrected.
